# Disulfidptosis: A Metabolic Cell Death Mechanism with Therapeutic Potential in Cancer

**DOI:** 10.32604/or.2026.076406

**Published:** 2026-03-23

**Authors:** Wubin Zhao, Qi Wang, Jun Zhang

**Affiliations:** Department of Spine Surgery, Pingdingshan First People’s Hospital, Pingdingshan, China

**Keywords:** Regulated cell death, disulfidptosis, solute carrier family 7 member 11, tumor metabolism, cancer therapy

## Abstract

Disulfidptosis is a newly identified form of regulated cell death (RCD) first described in 2023, representing a significant advance in understanding programmed cell death pathways. This unique cell death modality is characterized by abnormal intracellular accumulation of disulfide bonds and disruption of redox homeostasis, leading to cytoskeletal collapse without caspase activation. Disulfidptosis is primarily triggered by glucose deprivation in cells with high expression of solute carrier family 7 member 11 (SLC7A11). Under these conditions, insufficient NADPH supply prevents the effective reduction of accumulated cystine to cysteine, thereby inducing disulfide stress. Distinct from apoptosis, ferroptosis, cuproptosis, or pyroptosis, disulfidptosis exhibits unique metabolic dependencies and a hallmark feature of cytoskeletal disintegration. Current evidence indicates that this mechanism is operative in various tumor types, including hepatocellular carcinoma, colorectal cancer, and lung adenocarcinoma, suggesting its potential therapeutic relevance. Therapeutic strategies targeting disulfidptosis include modulation of metabolic pathways—such as the use of GLUT1 or G6PD inhibitors—to selectively induce this form of cell death in cancer cells. This review systematically summarizes current understanding, aiming to elucidate the unique mechanisms and therapeutic potential of disulfidptosis, and provides a foundational framework for future studies and the development of innovative strategies targeting tumor metabolic vulnerabilities.

## 1 Introduction and Overview of Disulfidptosis

Regulated cell death (RCD) refers to forms of cell death that are controlled by specific molecular pathways and can be modulated through genetic or pharmacological interventions, with its dysregulation being causally linked to various diseases, including cancer [1]. Given that cancer cells often develop drug resistance by evading cell death mechanisms [2], exploring alternative RCD mechanisms beyond traditional apoptosis is of significant importance for cancer therapy. In 2023, a novel form of cell death—disulfidptosis—was first identified and named [3]. Its initial discovery was based on the specific death of solute carrier family 7 member 11 (SLC7A11)-overexpressing cells under glucose deprivation, a form of cell death distinct from previously known types. The core mechanism of disulfidptosis lies in the abnormal accumulation of intracellular disulfide bonds and the disruption of redox homeostasis, which subsequently affects protein structure and function, ultimately leading to cell death. This form of cell death is independent of caspase activation; instead, it occurs through disulfide bond-mediated actin cross-linking that disrupts the cytoskeleton—mechanistically distinct from apoptosis (triggered by caspase activation) and ferroptosis (driven by the accumulation of lipid peroxides) [4,5]. Current studies indicate that disulfidptosis primarily occurs in cells with high expression of SLC7A11, particularly under conditions of glucose deprivation [6]. This suggests that disulfidptosis is not universally present in all cell types, but rather exhibits certain cell-type specificity. Moreover, studies have shown that lactate dehydrogenase B (LDHB) promotes disulfidptosis and thereby mediates the exhaustion of tumor-infiltrating CD8+ T cells, revealing the role of disulfidptosis in immune cells and its impact on the tumor microenvironment [7]. These findings indicate that disulfidptosis plays a crucial role in tumors. Beyond disulfidptosis, tumors also exhibit pronounced metabolic reprogramming. Within the tumor microenvironment, cancer cells display dependencies on specific nutrients or metabolic pathways. Targeting these aberrant metabolic features offers high specificity, rendering disulfidptosis a promising strategy for tumor-targeted therapy [8,9]. This is manifested as a dependency on specific nutrients or metabolic pathways. Targeting the aberrant metabolism of tumor cells offers high specificity, making disulfidptosis a promising strategy for tumor-targeted therapy [10,11].

In summary, studying disulfidptosis in the context of cancer holds significant importance. Given that many cancer cells can evade therapy-induced apoptosis, leading to drug resistance and disease recurrence [12,13], targeting disulfidptosis opens new avenues for developing novel cancer therapeutic strategies. Its unique metabolic sensitivity and impact on the actin cytoskeleton make it a promising target for cancer therapy.

The core objective of this review is to provide a timely and comprehensive synthesis of research on “disulfidptosis”—an emerging form of cell death—in the context of cancer. To this end, the article first aims to thoroughly elucidate its underlying molecular mechanisms, followed by a systematic summary of how disulfidptosis is regulated and functions across different tumor types. By comparing it with other cell death pathways, the review further clarifies its unique characteristics. Building upon this foundation, it consolidates current potential therapeutic strategies aimed at inducing disulfidptosis. Finally, by highlighting key unresolved questions in the field, the review offers a forward-looking perspective to guide future basic research and clinical translation efforts.

## 2 Molecular Mechanisms of Disulfidptosis

**Figure 1:**
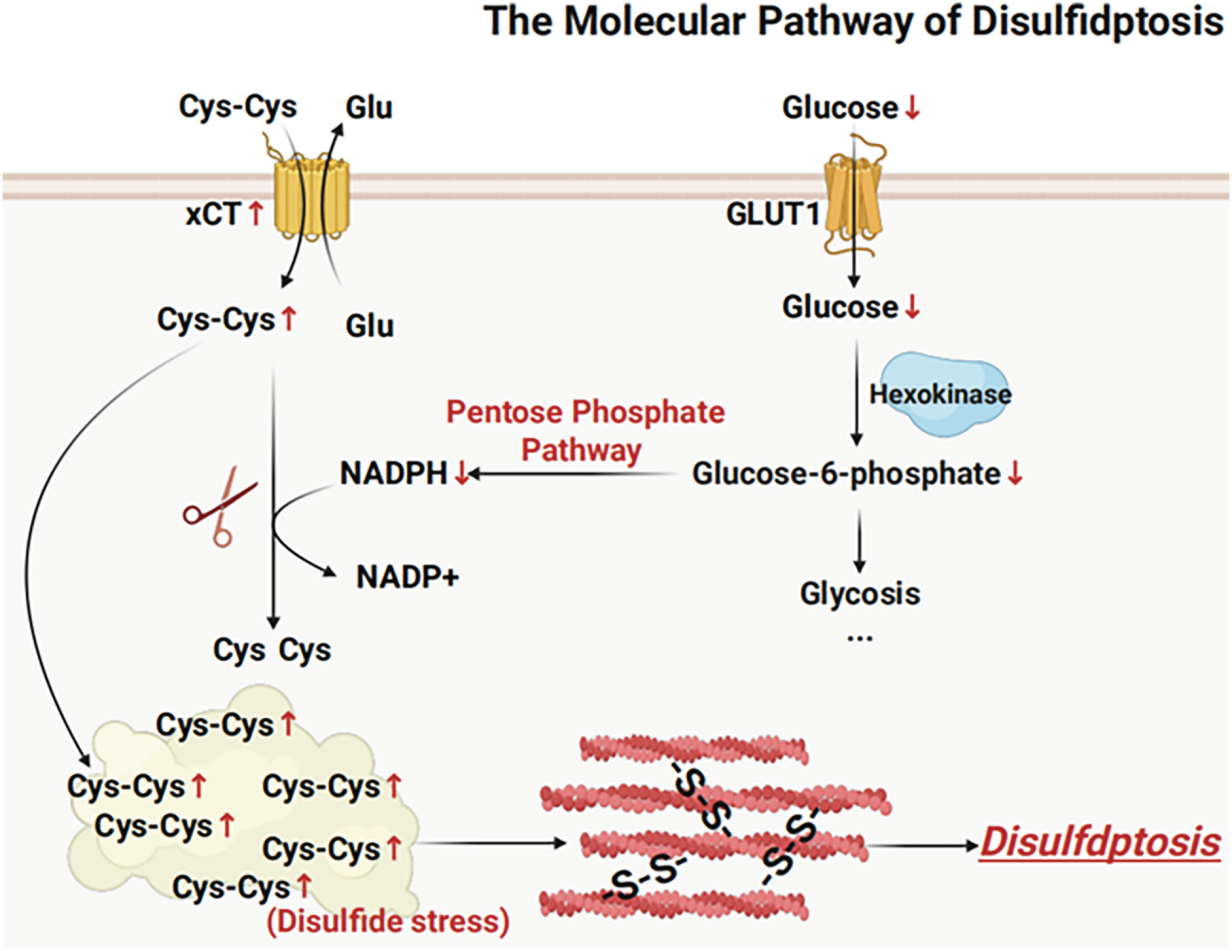
Basic mechanisms of disulfidptosis. Disulfidptosis is a novel form of cell death mediated by solute carrier family 7 member 11, which encodes the xCT transporter protein. Under glucose deprivation, cells with high SLC7A11 expression excessively import cystine. Due to insufficient NADPH supply, cystine cannot be efficiently reduced to cysteine, leading to abnormal accumulation of intracellular disulfides. This disulfide stress induces aberrant disulfide bonding and contraction within the actin cytoskeleton, ultimately resulting in cytoskeletal collapse and cell death. Abb: Glutamate (Glu), Cysteine (Cys), Cystine/Glutamate Antiporter (Xct) (Created with BioRender.com).

Disulfidptosis, as a novel form of programmed cell death, involves complex and intricate molecular mechanisms. It encompasses the synergistic actions of multiple critical metabolic pathways and organelles, collectively constructing a comprehensive pathway from initial triggers to cell death [14]. The core of this process lies in the vulnerability of cancer cells in maintaining redox homeostasis, and the induction of catastrophic disulfide stress under specific metabolic pressures [3]. Fig. 1 provides a brief summary of the molecular mechanisms of disulfidptosis.

### 2.1 Triggers of Disulfidptosis: Glucose Deprivation and High Expression of SLC7A11

Disulfidptosis is initiated under specific conditions, including glucose deprivation and high expression of SLC7A11 [14,15]. Activating transcription factor 4 (ATF4) and nuclear factor erythroid 2-related factor 2 (NRF2) are two key factors responsible for activating SLC7A11 transcription [16]. Glucose deprivation promotes ATF4 translation by inducing endoplasmic reticulum stress. Glucose is a crucial source for cellular energy metabolism. In oral squamous cell carcinoma cells, glucose deprivation significantly enhances ATF4 enrichment in the SLC7A11 promoter region and upregulates SLC7A11 mRNA levels [17]. Under oxidative stress conditions, NRF2 binds to the antioxidant response element (ARE) in the SLC7A11 promoter region, thereby markedly enhancing its transcriptional activity [18]. When cells are under glucose deprivation, the glycolytic pathway is impaired, leading to reduced generation of NADPH [19]. NADPH is a crucial intracellular reductant that provides electrons for glutathione (GSH) reductase, thereby maintaining cellular redox homeostasis. Under conditions of insufficient NADPH supply, cells are unable to effectively eliminate excess reactive oxygen species (ROS) and disulfide bonds, leading to oxidative stress and disulfide stress [20–22]. SLC7A11, also known as xCT, is a cystine/glutamate antiporter primarily responsible for importing extracellular cystine into the cell [19]. After entering the cell, cystine is reduced to cysteine, which serves as a key precursor for the synthesis of GSH. GSH is the primary intracellular antioxidant that neutralizes ROS and alleviates oxidative stress. However, under glucose deprivation, high expression of SLC7A11 leads to continuous cystine uptake, further depleting the already limited NADPH pool. This exacerbates the accumulation of disulfide bonds, ultimately triggering disulfidptosis [19,23].

### 2.2 Disulfide Stress

The redox balance of cells relies on the precise regulation of multiple factors, among which nicotinamide adenine dinucleotide phosphate (NADPH) plays a crucial role [24–28]. NADPH is primarily generated through the pentose phosphate pathway (PPP) [29]. It is the primary intracellular reductant, playing a key role in maintaining reduced GSH levels, scavenging ROS, and participating in various anabolic reactions [30]. The STAT3–LDHB–G6PD signaling axis has been identified as a key pathway regulating disulfidptosis and exhaustion in CD8^+^ T cells, highlighting the important role of G6PD in mediating disulfidptosis [7].

In tumor cells with high expression of SLC7A11, the cells take up large amounts of cystine and convert it into cysteine [23]. This process requires the consumption of large amounts of NADPH [19]. Under glucose deprivation conditions, the supply of NADPH in tumor cells is severely limited, leading to a disruption of the intracellular redox balance [15]. The depletion of NADPH directly impairs the reduction of disulfide bonds, causing intracellular disulfide bonds to fail to be properly reduced and leading to their abnormal accumulation [3]. This abnormal accumulation of disulfide bonds particularly affects proteins sensitive to redox state, such as actin cytoskeletal proteins, severely impairing their structure and function [23].

Therefore, NADPH depletion and the abnormal accumulation of disulfide bonds constitute the core mechanism underlying disulfidptosis [31]. On one hand, glucose deprivation limits NADPH production; on the other hand, excessive cystine uptake in tumor cells with high SLC7A11 expression further exacerbates NADPH consumption. This dual stress leads to intracellular redox imbalance and a burst of disulfide stress, ultimately triggering disulfidptosis. The following sections will further explore how this stress affects the cytoskeleton and the roles of other regulatory factors in this process.

### 2.3 Effector: Collapse of the Actin Cytoskeleton

Disulfidptosis, as a novel form of cell death triggered by disulfide stress, is characterized prominently by the collapse of the actin cytoskeleton [32]. The integrity of the cytoskeleton is crucial for maintaining cell morphology, motility, and intracellular transport [33]. Disulfidptosis exerts its cytotoxic effects precisely by disrupting the actin cytoskeleton. Under conditions such as glucose deprivation, cells with high SLC7A11 expression abnormally accumulate disulfides, leading to the formation of aberrant disulfide bonds within actin cytoskeletal proteins [3].

Specifically, the reduction state of intracellular cysteine residues is tightly regulated to maintain normal protein function [34–36]. However, during disulfidptosis, this balance is disrupted, leading to excessive formation of disulfide bonds within actin monomers and other cytoskeleton-associated proteins. These aberrant disulfide bonds interfere with the normal assembly and polymerization of actin, causing F-actin depolymerization and consequently disrupting the structural integrity of the entire cytoskeleton [3].

The collapse of the cytoskeleton directly impairs cell morphology and function. For instance, cells lose their normal shape, exhibit reduced adhesion, and impaired migration. More importantly, disruption of the cytoskeleton also interferes with intracellular signaling and transport, ultimately leading to cell death. This phenomenon is particularly evident in cancer cells with high SLC7A11 expression, especially when glucose uptake is limited [31,37,38].

Beyond direct structural damage, the collapse of the actin cytoskeleton may promote cell death through additional pathways. For example, the cytoskeleton interacts with receptors and ion channels on the cell membrane, modulating their activity. Disruption of the cytoskeleton could lead to dysfunction of these receptors and ion channels, further exacerbating cellular damage. Moreover, the cytoskeleton is involved in the regulation of processes such as autophagy and apoptosis. Therefore, cytoskeletal collapse may indirectly promote cell death by interfering with these pathways.

In summary, the collapse of the actin cytoskeleton is one of the key effectors of disulfidptosis. By forming aberrant disulfide bonds in actin and other cytoskeletal proteins, disulfidptosis rapidly disrupts the structure and function of the cytoskeleton, ultimately leading to cell death [32,39,40]. Understanding the molecular mechanisms of this process is crucial for developing anti-tumor therapeutic strategies that target disulfidptosis.

### 2.4 Other Regulatory Factors: WAVE Complex and Rac1

**Figure 2:**
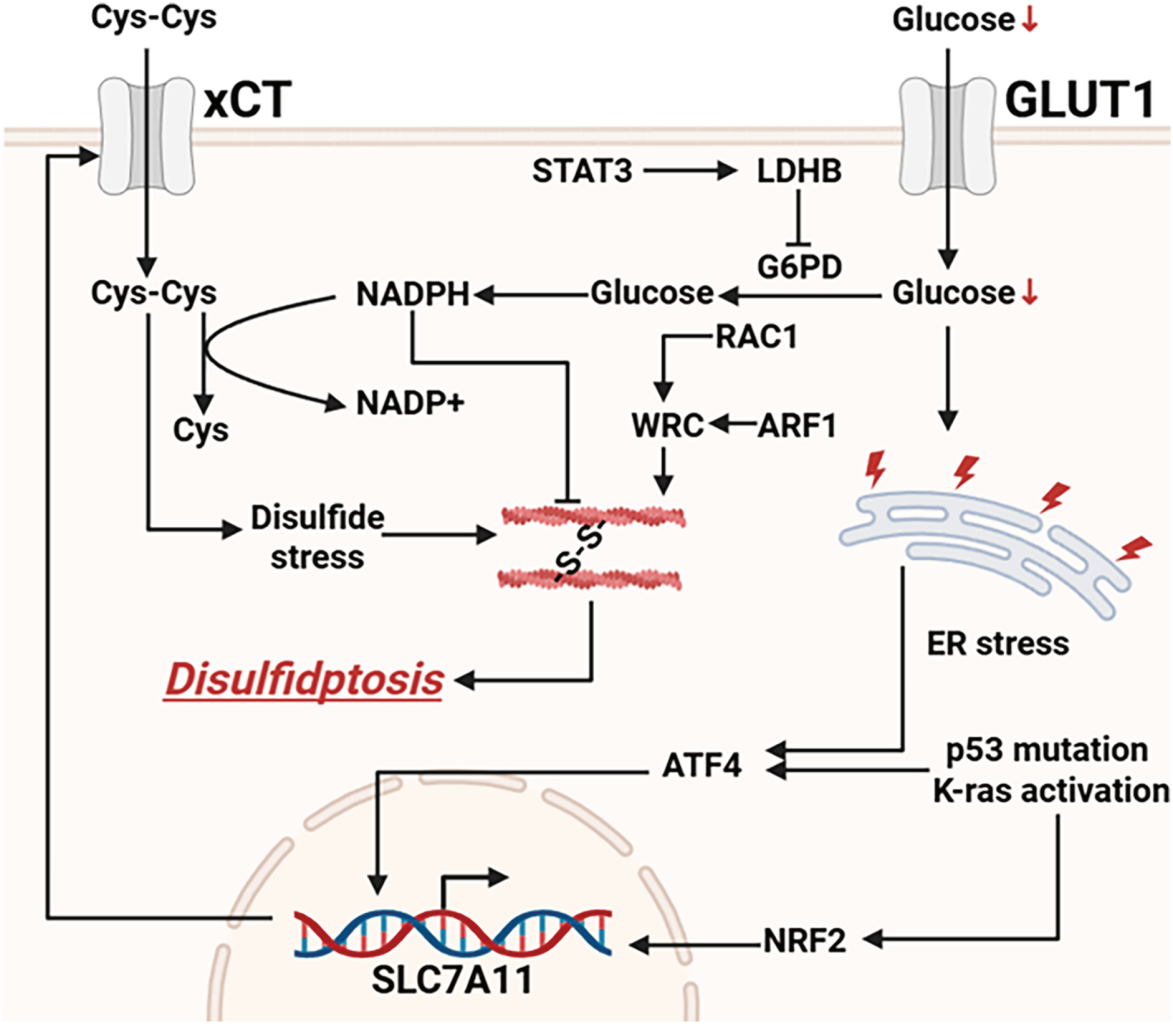
Currently known signaling pathways regulating disulfidptosis. LDHB inhibits NADPH production by suppressing G6PD. The transcription factors ATF4 and NRF2 can translocate into the nucleus to promote the transcription and expression of SLC7A11. Abb: Glucose Transporter 1 (GLUT1), Signal Transducer and Activator of Transcription 3 (STAT3), Lactate Dehydrogenase B (LDHB), Glucose-6-Phosphate Dehydrogenase (G6PD), Ras-related C3 botulinum toxin substrate 1 (RAC1), WAVE Regulatory Complex (WRC), ADP-Ribosylation Factor 1 (ARF1), Activating Transcription Factor 4 (ATF4), Nuclear factor erythroid 2–related factor 2 (NRF2), Solute Carrier Family 7 Member 11 (SLC7A11) (Created with BioRender.com).

In addition to the two key triggering conditions—glucose deprivation and high SLC7A11 expression—the WAVE regulatory complex (WRC) and the Rac1 pathway also play important roles in regulating disulfidptosis (Fig. 2). These molecules are involved in the process of actin polymerization, thereby influencing cytoskeletal stability and ultimately determining the cell’s sensitivity to disulfidptosis [39].

The WRC is a multi-protein complex essential for actin polymerization and involved in the formation of lamellipodia. Studies have shown that inactivation of the WRC can suppress disulfidptosis [41,42]. This may be because WRC function is critical for maintaining normal actin dynamics, and its inactivation weakens the cell’s ability to cope with disulfide stress in the actin cytoskeleton. On the other hand, constitutive activation of the small GTPase Rac1 promotes disulfidptosis [43]. Rac1 is an upstream activator of the WRC [44]. It influences actin polymerization by regulating WRC activity. Therefore, activation of Rac1 may enhance disulfide bond formation within the actin network, making it more prone to collapse and thereby promoting disulfidptosis. Additionally, Arf1 acts as a WRC activator and can synergize with Rac1 to promote WRC-mediated disulfidptosis; however, the precise mechanism of this activation remains unclear, and thus this pathway is not discussed in detail in this review [45].

In summary, WRC and Rac1 modulate cellular sensitivity to disulfidptosis by regulating actin polymerization. Inactivation of WRC suppresses actin polymerization and thereby inhibits disulfidptosis, whereas activation of Rac1 promotes both actin polymerization and disulfidptosis. Targeting the Rac1-WRC signaling pathway may represent a novel strategy for modulating disulfidptosis in tumor cells.

## 3 Regulation and Roles of Disulfidptosis in Different Tumor Types

### 3.1 Pan-Cancer Analysis: The Universality and Specificity of Disulfidptosis-Related Genes

Pan-cancer analysis studies have generally revealed that disulfidptosis-related genes (DRGs) exhibit significant expression abnormalities across multiple tumor types [46,47]. These differences are not only evident between tumor tissues and normal tissues, but also exist among different tumor subtypes [48]. For example, analysis of 15 DRGs, including SLC7A11, INF2, and CD2AP, revealed significant differences in the expression levels of these genes across more than 9000 samples from over 30 cancer types [48]. Specifically, high expression of ACTB, ACTN4, and MYL6 in GBMLGG, LGG, MESO, and LAML is associated with poor prognosis, while high expression of INF2 in LIHC, LUAD, UVM, HNSC, GBM, LAML, and KIPAN is also linked to poor prognosis [49]. Moreover, as a key gene mediating disulfidptosis, SLC7A11 is significantly upregulated in 25 out of 27 tumor types in a pan-cancer analysis, highlighting the widespread activation of disulfidptosis [50]. Another pan-cancer study similarly reported broad upregulation of this gene across tumors [51]. Disulfidptosis has been more intensively studied across various tumors. Table 1 summarizes the mechanisms and pathways of disulfidptosis in different tumor types.

**Table 1:** Key molecules and mechanisms of disulfidptosis in various tumors.

Types of Tumors	Key Molecules or Pathways of Disulfidptosis	Functions of Key Molecules	References
Hepatocellular Carcinoma	MYH9	High expression is associated with poor survival; inhibiting MYH9 can alleviate sorafenib resistance by inducing disulfidptosis	[54]
Hepatocellular Carcinoma	GYS1	Associated with increased risk of HBV-HCC; silencing GYS1 suppresses tumor proliferation and metastasis; positively correlated with immune infiltration and microsatellite instability	[56]
Colorectal Cancer	DPP7	High expression is associated with poor prognosis; suppresses disulfidptosis and immune evasion through a GPX4-dependent mechanism; its depletion enhances NK cell-mediated tumor killing	[59]
Gastric Cancer	SLC7A11	Regulates cell death and influences gastric cancer progression; involved in the process of disulfidptosis	[60]
Gastric Cancer	NCKAP1	Overexpressed in GC, associated with actin activity, GTPase energy metabolism, immune infiltration, and immunotherapy response; a potential prognostic biomarker	[62]
Pancreatic Ductal Adenocarcinoma	CASC8	Highly expressed in PDAC and associated with poor prognosis; enhances c-Myc stability through interaction, activating the pentose phosphate pathway and inhibiting disulfidptosis under glucose starvation	[63]
Pancreatic Ductal Adenocarcinoma	MET	Significantly positively correlated with disulfidptosis and influences the invasion and metastasis of PDAC	[64]
Ovarian Cancer	GYS1	The rate-limiting enzyme for glycogen synthesis, promotes NADPH production through the p53/GYS1 positive feedback loop, counteracts disulfidptosis, and leads to platinum resistance	[65]
Prostate Cancer	CCNB2	A novel regulator of disulfidptosis; its downregulation promotes disulfidptosis and inhibits migration and proliferation of prostate cancer cells; the Dis score is associated with its expression and predicts prognosis	[66]
Bladder Cancer	GLUT1/Cystine-Containing Polymers	Nano-sonodynamic therapy inhibits GLUT1 and releases cystine to induce disulfidptosis, promotes immunogenic cell death, and synergizes with PD-1 antibodies for antitumor effects	[68]
Breast Cancer	Disulfidptosis-Related lncRNA	Construct prognostic models to differentiate breast cancer subtypes, predict outcomes, and guide treatment	[70]
Lung Adenocarcinoma	ZIC5	High expression is associated with poor prognosis; silencing ZIC5 inhibits proliferation and energy metabolism while promoting disulfidptosis, indicating its potential as a therapeutic target	[72]
Lung Adenocarcinoma	NCKAP1	High expression is associated with poor prognosis and immune evasion; targeting NCKAP1 may enhance immunotherapy response	[74]
Lung Adenocarcinoma	Disulfidptosis-Related lncRNA	Construct prognostic models to predict survival, TMB, immune infiltration, and response to immunotherapy/targeted therapy	[75]
Glioma	Disulfidptosis-related gene risk score	High-risk scores are associated with specific immune cell infiltration patterns and elevated expression of immune checkpoints and chemokines	[76]
Glioma	Endothelial cell disulfidptosis-Tex pathway	Endothelial cells exhibit high disulfidptosis-T cell exhaustion activity, and memory T cells are associated with this gene activity	[77]
Melanoma	FTO	Elevated expression leads to decreased m6A levels and promotes tumor invasion; the FTO inhibitor MA can restore m6A levels, upregulate SLC7A11, and induce disulfidptosis	[78]
Head and Neck Squamous Cell Carcinoma	PPARγ	Inhibiting PPARγ upregulates HMOX1 and SLC7A11, promoting ferroptosis and disulfidptosis respectively, and synergistically exerts antitumor effects	[79]

Note: Abb: Myosin Heavy Chain 9 (MYH9), Hepatocellular Carcinoma (HCC), Glycogen Synthase 1 (GYS1), Dipeptidyl Peptidase 7 (DPP7), Glutathione Peroxidase 4 (GPX4), Natural Killer (NK), Solute carrier family 7 member 11 (SLC7A11), Gastric Cancer (GC), NCK-Associated Protein 1 (NCKAP1), Cancer Susceptibility Candidate 8 (CASC8), Mesenchymal–Epithelial Transition factor (MET), Pancreatic Ductal Adenocarcinoma (PDAC), Glycogen Synthase 1 (GYS1), Cyclin B2 (CCNB2), Glucose Transporter 1 (GLUT1), Programmed Cell Death Protein 1 (PD-1), Zinc Finger Protein of the Cerebellum 5 (ZIC5), Fat Mass and Obesity-Associated Protein (FTO), Heme Oxygenase 1 (HMOX1), Peroxisome Proliferator-Activated Receptor Gamma (PPARγ).

### 3.2 Disulfidptosis in Gastrointestinal Tumors

#### 3.2.1 Hepatocellular Carcinoma (HCC)

In HCC, multi-omics analysis has been employed to explore the disulfidptosis landscape and establish a new model for predicting the prognosis of HCC patients [52]. Machine learning has also been used to identify disulfidptosis subtypes in HCC [53]. Based on the transcriptomic profiles of 31 disulfidptosis-related genes, HCC patients were stratified into two groups: C1, characterized by high disulfidptosis activity, and C2, characterized by low disulfidptosis activity. The C2 group, with lower disulfidptosis levels, generally exhibited better overall survival (OS) and progression-free survival (PFS), reduced infiltration of immunosuppressive cells, and activation of the glycine/serine/threonine metabolism pathway [53]. In mechanistic studies, research has found that high expression of disulfidptosis-related genes is associated with poor survival in liver cancer patients [54]. The MYH9 gene plays a key role, and inhibition of MYH9 can alleviate sorafenib resistance in HCC through disulfidptosis-like changes [54]. This provides a new strategy for enhancing tumor cell sensitivity to drugs. Additional evidence has revealed that the disulfidptosis-related gene *RPN1* is markedly upregulated in both HCC tissues and peripheral blood mononuclear cells (PBMCs) from HCC patients, with its expression levels correlating positively with disease progression. Functional assays showed that RPN1 overexpression suppresses HCC cell proliferation, migration, and tumorigenicity *in vivo*, whereas RPN1 knockdown exerts the opposite effects. Mechanistically, RPN1 induces G0/G1 cell cycle arrest by modulating key cell cycle regulators—including CDK1, CDK2, Cyclin D1, and Cyclin E1—thereby impeding tumor progression [55]. These findings collectively suggest that RPN1 may serve as a potential therapeutic target and prognostic biomarker in HCC.

Regarding HBV-related hepatocellular carcinoma (HBV-HCC), studies have shown that increased genetic susceptibility to HBV increases the risk of developing HCC. Moreover, genetic variations in disulfidptosis are significantly associated with an increased risk of HBV-HCC [56]. GYS1 is considered a potential therapeutic target for HBV-HCC, showing a significant positive correlation with immune infiltration and microsatellite instability (MSI). Silencing GYS1 can effectively inhibit tumor proliferation and metastasis in HBV-HCC [56].

#### 3.2.2 Colorectal Cancer (CRC)

In the field of CRC, research on disulfidptosis primarily focuses on constructing prognostic models, evaluating the immune microenvironment, and exploring potential therapeutic targets. Studies generally suggest that disulfidptosis is closely associated with the prognosis of CRC patients [57,58], and it may act as a protective factor for CRC patients. Dipeptidyl peptidase 7 (DPP7) promotes colorectal cancer progression by inhibiting disulfidptosis and NK cell-mediated cytotoxicity in a GPX4-dependent manner. DPP7 overexpression reduces glucose deprivation-induced disulfide bond formation in cytoskeletal proteins (drebrin, FLNA, FLNB), protecting cells from disulfidptosis. Mechanistically, DPP7 physically interacts with and stabilizes GPX4 protein without affecting its mRNA. Restoring GPX4 in DPP7-depleted cells rescues resistance to disulfidptosis and immune killing, while GPX4 knockdown abolishes DPP7’s protective effect [59]. High expression of DPP7 is associated with poor prognosis in CRC patients, while the depletion of DPP7 can enhance the cytotoxicity of natural killer (NK) cells against tumor cells [59].

#### 3.2.3 Gastric Cancer(GC)

GC, as a common malignant tumor of the digestive system, is characterized by poor prognosis and high mortality. Studies have shown that SLC7A11 can regulate cell death and influence the progression of GC, suggesting that disulfidptosis may be involved in the pathological process of GC [60]. Studies have found that several disulfidptosis-related genes may be associated with the prognosis of GC, and a predictive model incorporating multiple genes has been constructed [61]. Clinical studies have shown that NCKAP1 and SLC7A11 are overexpressed in GC, and they are associated with actin activity, GTPase energy metabolism, immune infiltration, and immunotherapy [62]. These genes can serve as potential prognostic and diagnostic biomarkers for GC.

#### 3.2.4 Pancreatic Ductal Adenocarcinoma (PDAC)

PDAC is a highly lethal malignancy. Studies have confirmed the presence of disulfidptosis in PDAC, and it has been found that the oncogene CASC8 plays a role in this process [63]. CASC8 is expressed at higher levels in PDAC tissues compared to normal pancreatic tissues, and high CASC8 expression is associated with poor prognosis in PDAC patients [63]. CASC8 interacts with c-Myc, enhancing the stability of the c-Myc protein and activating the pentose phosphate pathway, which lowers the NADP/NADPH ratio, thereby inhibiting disulfidptosis under glucose-starved conditions [63]. The study also constructed a prognosis-related signature associated with disulfidptosis, and found that the MET gene is significantly positively correlated with disulfidptosis and influences the invasion and metastasis of PAAD [64].

### 3.3 Disulfidptosis in Genitourinary Tumors

#### 3.3.1 Disulfidptosis in Ovarian Cancer

Ovarian clear cell carcinoma exhibits unique metabolic characteristics, demonstrating higher glycogen levels and resistance to platinum-based drugs [65]. Research has found that the glycogen synthesis rate-limiting enzyme GYS1 is associated with poor prognosis and chemotherapy resistance in OCCC. It promotes the production of energetic NADPH through a p53/GYS1 positive feedback loop, thereby resisting disulfidptosis and enhancing OCCC’s resistance to platinum-based drugs [65].

#### 3.3.2 Disulfidptosis in Prostate Cancer and Renal Cell Carcinoma

In the field of prostate cancer, CCNB2 has been identified as a novel regulator of disulfidptosis. A molecular subtyping framework based on disulfidptosis was constructed to classify prostate cancer into distinct subtypes, and the Dis score was used to assess the severity of each patient subtype. The results indicate that the Dis score is significantly associated with PCa prognosis, with higher Dis scores correlating with lower tumor mutational burden and improved outcomes [66]. Experimental results confirmed that downregulation of CCNB2 expression promotes disulfidptosis in prostate cancer cells, thereby inhibiting their migration and proliferation capabilities [66]. In clear cell renal cell carcinoma (ccRCC), a recent study similarly established a novel classification system—termed the Disulfidptosis-based Classifier System (DCS)—based on disulfidptosis-related genes, stratifying ccRCC into four distinct molecular subtypes. Among these, the DCS3 subtype exhibits hallmark features of suppressed disulfidptosis, insensitivity to immunotherapy, pronounced genomic instability, and poor clinical outcomes. Notably, the study further demonstrated that NU1025 significantly inhibits the malignant progression of DCS3-subtype tumors, thereby offering a promising strategy for precision therapy in ccRCC [67].

#### 3.3.3 Disulfidptosis in Bladder Cancer

In bladder cancer, the combination of nanotechnology and sonodynamic therapy, utilizing GLUT1 inhibitors and cystine-containing polymers to induce disulfidptosis, has demonstrated improved efficacy in immunotherapy [68]. This approach effectively releases Bay-876, disrupts intracellular redox homeostasis, releases cystine to induce disulfidptosis, promotes immunogenic cell death, and synergizes with PD-1 monoclonal antibodies to suppress tumor growth [68].

### 3.4 Disulfidptosis in Breast Cancer and Lung Cancer.

#### 3.4.1 Disulfidptosis in Breast Cancer

Breast cancer is a highly heterogeneous disease, classified into molecular subtypes such as Luminal A, Luminal B, HER2-positive, and Basal-like. Emerging research indicates that disulfidptosis plays a complex and critical role in the development and progression of breast cancer [69].

Long non-coding RNAs play a crucial role in gene expression regulation. Some studies have attempted to construct predictive models based on disulfidptosis-related lncRNAs to differentiate between molecular subtypes of breast cancer [70]. These models can help predict breast cancer prognosis and provide guidance for clinical treatment.

#### 3.4.2 Disulfidptosis in Lung Cancer

Lung cancer is one of the leading causes of cancer-related deaths worldwide, with lung adenocarcinoma being the most common subtype. Growing evidence suggests that disulfidptosis plays a significant role in the development and progression of lung adenocarcinoma [71]. ZIC5, a member of the Zic family of transcription factors, is highly expressed in lung adenocarcinoma cells and is associated with poor patient prognosis [72]. Silencing ZIC5 suppresses proliferation and energy metabolism in lung adenocarcinoma cells while promoting disulfidptosis [72]. This suggests that targeting ZIC5 may represent a novel therapeutic strategy for lung adenocarcinoma. NCKAP1, a disulfidptosis-related gene, shows significant differential expression across various cancers [73]. In lung adenocarcinoma, high expression of NCKAP1 is associated with poor prognosis and immune evasion mechanisms [74]. Targeting NCKAP1 may enhance the response of lung adenocarcinoma patients to immunotherapy. Similar to breast cancer, researchers have also developed prognostic models for lung adenocarcinoma based on disulfidptosis-related lncRNAs [75]. These models can not only predict patient survival but also forecast tumor mutational burden, immune cell infiltration, and responses to immunotherapy and targeted therapy.

### 3.5 Disulfidptosis in Other Tumors

Disulfidptosis may influence the immune microenvironment in glioma. Studies have found that glioma patients with high-risk scores exhibit distinct immune cell infiltration patterns, and most immune checkpoints and chemokines show a positive correlation with risk scores [76]. Furthermore, studies have revealed elevated disulfidptosis-T cell exhaustion activity in glioma endothelial cells, and memory T cell populations are associated with these genes [77].

Melanoma is a malignant tumor originating from melanocytes, with uveal melanoma being the most common primary intraocular malignancy in adults and associated with an extremely poor prognosis. Research indicates that N6-methyladenosine modification plays a critical role in the development and progression of melanoma, while FTO acts as an m6A demethylase. Elevated FTO expression in melanoma tissues is correlated with reduced m6A levels, increased tumor aggressiveness, and poor prognosis [78]. The FTO inhibitor meclofenamic acid (MA) has been shown to restore m6A RNA methylation levels in uveal melanoma cells, leading to upregulation of SLC7A11 and triggering disulfidptosis, a form of cell death driven by glutathione depletion and NADPH consumption. *In vitro* and *in vivo* experiments demonstrated that MA treatment effectively induced disulfidptosis and suppressed tumor growth, supporting FTO inhibition as a potential therapeutic strategy [78].

In head and neck squamous cell carcinoma, studies indicate that PPARγ antagonists exert antitumor effects by modulating ferroptosis and disulfidptosis. In oral squamous cell carcinoma, PPARγ inhibition leads to upregulation of heme oxygenase 1, promoting ferroptosis, while also upregulating SLC7A11 to induce disulfidptosis [79].

### 3.6 Therapeutic Implications across Different Tumor Types

Based on current research, bladder cancer and ovarian clear cell carcinoma are the tumor types most likely to benefit from interventions targeting disulfidptosis. In bladder cancer, studies have successfully combined nanotechnology with sonodynamic therapy to deliver GLUT1 inhibitors, thereby directly creating the metabolic conditions that trigger disulfidptosis—such as glucose deprivation. This approach has been effectively integrated with immunotherapy (e.g., PD-1 antibodies), establishing a well-defined therapeutic paradigm [68]. Ovarian clear cell carcinoma (OCCC) represents an ideal therapeutic target for disulfidptosis-based strategies due to its distinctive dysregulation of glycogen metabolism. The key enzyme glycogen synthase 1 (GYS1) drives a positive feedback loop that enhances NADPH production, thereby conferring resistance to disulfidptosis. Targeting GYS1 directly disrupts this protective mechanism and overcomes platinum resistance in OCCC [65]. Moreover, lung adenocarcinoma (via targeting of ZIC5 or NCKAP1) and melanoma (through FTO inhibitors that upregulate SLC7A11) also demonstrate clear therapeutic potential. These targets have been shown to directly regulate disulfidptosis and are associated with poor prognosis, thereby offering well-defined avenues for drug development [72,74,78].

## 4 Associations between Disulfidptosis and Other Forms of Cell Death

**Figure 3:**
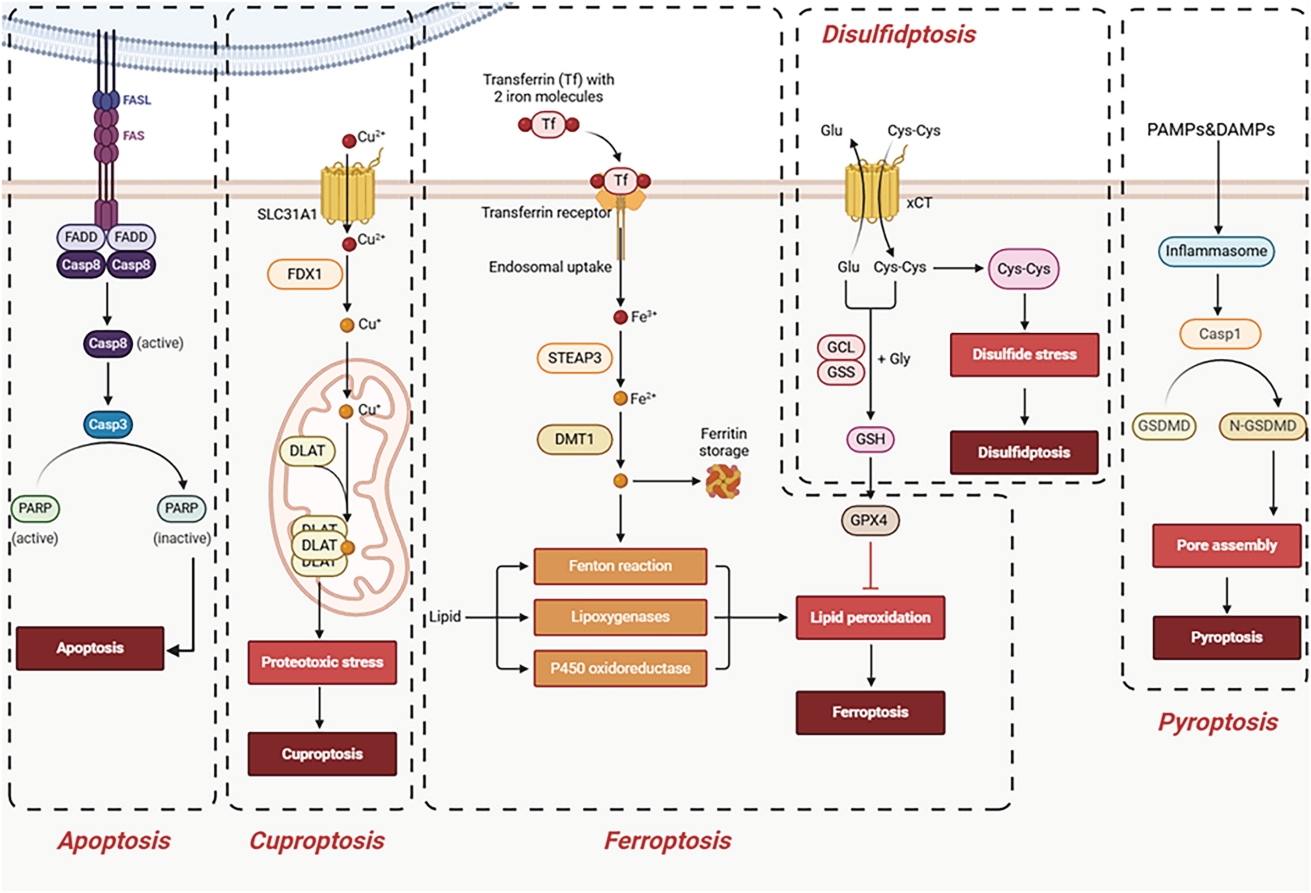
Associations between disulfidptosis and other forms of cell death. Unlike apoptosis, ferroptosis, cuproptosis, or pyroptosis, disulfidptosis is triggered by glucose starvation in SLC7A11-high cells, leading to disulfide stress and actin cytoskeleton collapse, independent of caspases, iron, copper, or inflammation. Abb: Fas-Associated Protein with Death Domain (FADD), Poly (ADP-Ribose) Polymerase (PARP), Ferredoxin (FDX), Dihydrolipoamide S-Acetyltransferase (DLAT), Six-Transmembrane Epithelial Antigen of the Prostate 3 (STEAP3), Divalent Metal Transporter 1 (DMT1), Glutamate–Cysteine Ligase (GCL), Glutathione Synthetase (GSS), Glutathione (GSH), Caspase 1 (Casp1) (Created with BioRender.com).

Cell death is a crucial process for maintaining homeostasis in multicellular organisms [80]. Historically, apoptosis was considered the primary programmed cell death pathway, but research in recent decades has shown that various regulated non-apoptotic cell death forms also play significant roles in disease development [81]. Disulfidptosis, as a newly discovered form of programmed cell death, is distinctly different from other known cell death pathways such as apoptosis, ferroptosis, cuproptosis, and pyroptosis [82–85]. This section will focus on comparing the similarities and differences between disulfidptosis and other programmed cell death pathways, highlighting its unique metabolic dependencies and cytoskeletal collapse characteristics, and exploring its potential implications in tumors (Table 2, Fig. 3).

**Table 2:** The distinction between disulfidptosis and other forms of cell death.

Characteristics	Apoptosis	Ferroptosis	Cuproptosis	Pyroptosis	Disulfidptosis
Key Regulators	Caspases	GPX4, Iron ions	Copper ions, lipid-acylated proteins	Caspase-1, Gasdermin D(GSDMD)	SLC7A11, glucose, actin cytoskeleton
Key Mechanisms	Caspase activation, DNA fragmentation, formation of apoptotic bodies	Lipid peroxidation	Copper ions bind to lipid-acylated proteins, causing mitochondrial dysfunction	GSDMD-mediated cell membrane pore formation, inflammation	SLC7A11, glucose starvation, disulfide bond accumulation, cytoskeleton collapse
Inflammatory	No	No	No	Yes	No
Metabolic Dependencies	Lower	Higher, dependent on iron metabolism	Higher, dependent on copper metabolism	Lower	Extremely high, dependent on glucose metabolism
Cytoskeletal Impact	Lower	Lower	Lower	Lower	Extremely high, actin cytoskeleton collapse
Reference	[100–103]	[92,94,104]	[97,105,106]	[99,107,108]	[32]

### 4.1 Apoptosis

Apoptosis is divided into intrinsic apoptosis and extrinsic apoptosis [86–88]. The intrinsic apoptotic pathway is typically triggered by intracellular stresses such as DNA damage, oxidative stress, or growth factor deprivation. This initially activates stress-sensing molecules like p53, which upregulates pro-apoptotic Bcl-2 family members such as Bax, Puma, and Noxa. These proteins promote the oligomerization of Bax/Bak in the outer mitochondrial membrane, forming transmembrane pores that lead to mitochondrial outer membrane permeabilization (MOMP) [89]. This process is accompanied by the release of pro-apoptotic factors such as cytochrome c and Smac/DIABLO into the cytosol, which alleviates the inhibitory effect of IAPs (Inhibitor of Apoptosis Proteins) on caspases [90]. Cytochrome c, together with Apaf-1 and the procaspase-9 precursor, assembles into a heptameric complex known as the “apoptosome,” which triggers the autocatalytic cleavage and activation of caspase-9 [90]. Activated caspase-9 subsequently initiates a cascade of effector caspases—caspase−3, −6, and −7—which cleave substrates such as PARP, gelsolin, and nuclear structural proteins, ultimately executing hallmark apoptotic events including cytoplasmic shrinkage, nuclear fragmentation, and internucleosomal DNA cleavage.The extrinsic pathway is triggered when ligands such as FasL or TNF-α bind to their respective death receptors, inducing receptor trimerization and recruitment of adaptor proteins FADD and TRADD along with procaspase-8 to form the Death-Inducing Signaling Complex (DISC) [91]. At the mechanistic level, disulfidptosis is a form of regulated cell death triggered by disulfide bond stress. Its core mechanism involves SLC7A11 overexpression combined with glucose restriction, which leads to NADPH depletion. This impairs the reduction of intracellular cystine, resulting in excessive accumulation of disulfide bonds. These aberrant disulfide bonds cause non-physiological crosslinking of cytoskeletal proteins such as actin, ultimately leading to collapse of the F-actin network and cell death. This process is further exacerbated through actin-remodeling pathways—such as the Rac–WRC–Arp2/3 axis—which promote additional disulfide bond formation [32]. Therefore, the execution mechanism of disulfidptosis is closely linked to the classic paradigm of “protein/cytoskeletal crosslinking + cellular structural disruption,” rather than relying on traditional pathways such as apoptosis, lipid peroxidation, or inflammatory membrane pore formation.

### 4.2 Ferroptosis

The core of ferroptosis lies in the uncontrolled accumulation of lipid peroxidation and the susceptibility of cellular membranes enriched with polyunsaturated fatty acids (PUFAs) [92]. Under normal conditions, GPX4 uses GSH as an electron donor to reduce membrane lipid hydroperoxides to their corresponding alcohols, thereby terminating the lipid peroxidation chain reaction. When GPX4 is inhibited or GSH is depleted, the cell loses its ability to reverse oxidative damage [93]. Meanwhile, ferrous iron (Fe²^+^) continuously generates hydroxyl radicals via the Fenton reaction, which initiates hydrogen abstraction from polyunsaturated fatty acid-containing phosphatidylethanolamines, thereby further driving the chain reaction of lipid peroxidation [10]. Lipid metabolism–related enzymes ACSL4 and LPCAT3 enhance cellular sensitivity to ferroptosis by selectively esterifying arachidonic acid and adrenic acid into membrane phospholipids [94]. Accumulation of peroxidized phospholipids disrupts the lipid-phase stability of cellular membranes, leading to impaired membrane curvature and abnormal membrane permeability. Characteristic ultrastructural features of ferroptosis include mitochondrial shrinkage, increased mitochondrial membrane density, and reduction or complete loss of cristae, while the nucleus and cytoskeleton remain largely intact—highlighting its distinct molecular and morphological differences from apoptosis, necrosis, and other forms of cell death [95]. Although both cuproptosis and disulfidptosis may involve disruption of cellular architecture or protein homeostasis, they differ fundamentally in their mechanisms and targets. Cuproptosis centers on the toxic aggregation of mitochondrial metabolic enzymes and Fe–S cluster proteins, leading to metabolic collapse, whereas disulfidptosis primarily involves disulfide-mediated cross-linking of cytoskeletal proteins and consequent physical disintegration of cellular structure. Consequently, these two forms of regulated cell death exhibit fundamental differences in their molecular targets, execution mechanisms, and physiological triggers [96].

### 4.3 Cuproptosis

Cuproptosis is a novel form of metabolism-associated regulated cell death, distinct from apoptosis, necrosis, and ferroptosis [97]. Its key event involves the intracellular accumulation of free copper ions, which bind with high affinity to lipoylated proteins in mitochondria—such as dihydrolipoamide S-acetyltransferase (DLAT) and dihydrolipoamide S-succinyltransferase (DLST)—triggering their aberrant aggregation and subsequent disruption of proteostasis [98]. This process inhibits key mitochondrial TCA cycle enzyme complexes—such as pyruvate dehydrogenase (PDH) and α-ketoglutarate dehydrogenase (KGDH)—leading to a sharp decline in energy metabolism. Concurrently, copper ions promote the destabilization of iron–sulfur (Fe–S) cluster-containing proteins, triggering oxidative stress and proteotoxic stress, which collectively impair the cell’s ability to maintain a reducing environment necessary for proper protein folding and homeostasis [98]. Together, these effects trigger a distinct form of cell death that depends on mitochondrial lipoylated proteins—fundamentally different from disulfidptosis, which is driven by NADPH depletion and the ensuing “disulfide storm.” In cuproptosis, copper exerts direct intrinsic toxicity by binding to and aggregating lipoylated proteins, whereas in disulfidptosis, loss of reducing capacity (due to NADPH exhaustion) leads to pathological accumulation of disulfide bonds [14].

### 4.4 Pyroptosis

Pyroptosis is an inflammatory form of programmed cell death, typically mediated by inflammasome-activated caspase-1, leading to cleavage of the GSDMD protein and the formation of cell membrane pores [99]. Pyroptosis is typically accompanied by the massive release of inflammatory factors. Disulfidptosis does not depend on inflammasome activation or GSDMD cleavage and is not an inflammatory form of cell death.

## 5 Strategies for Targeting Disulfidptosis in Tumor Therapy

**Figure 4:**
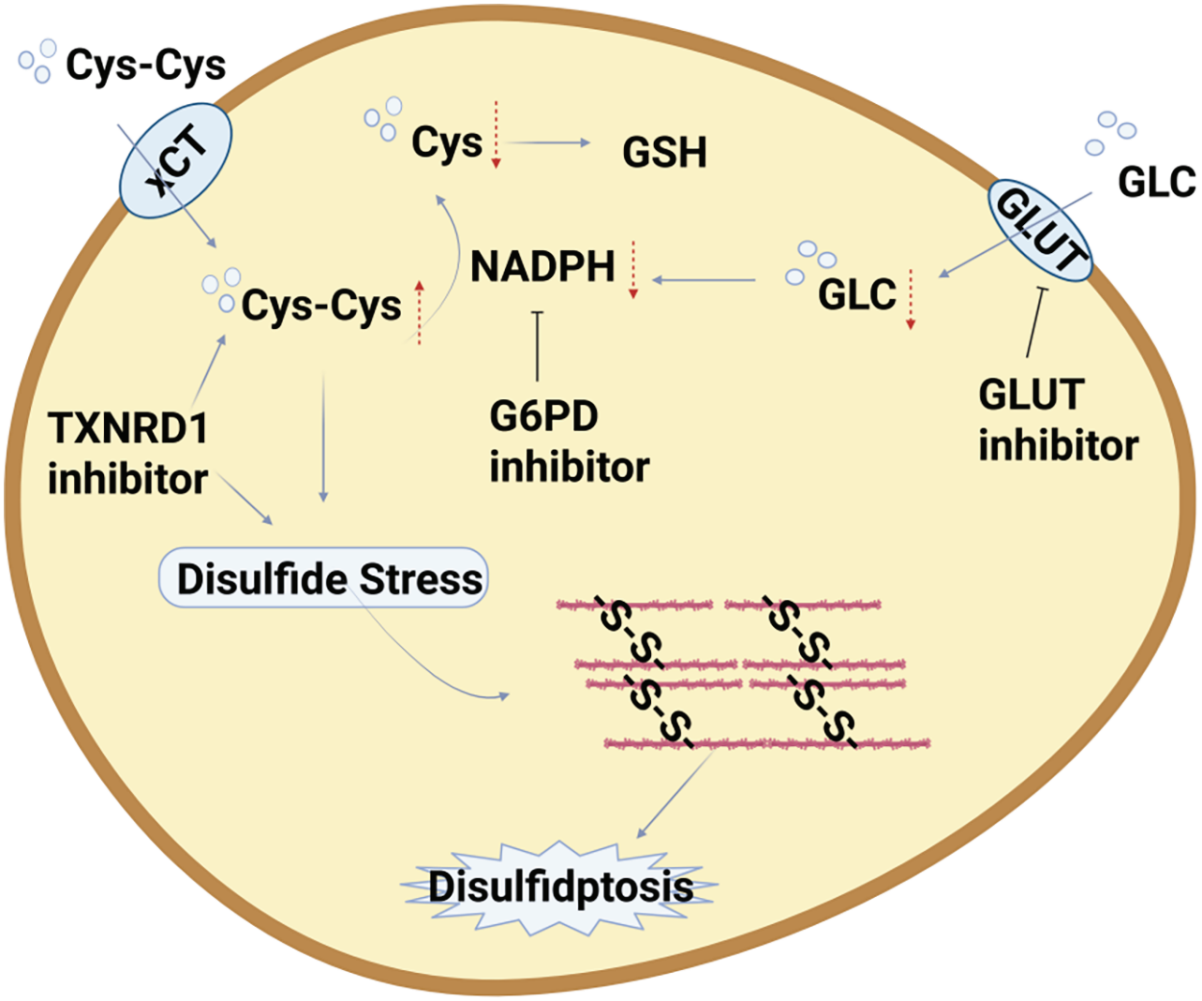
Strategies for Targeting the Disulfidptosis Pathway in Tumor Therapy. Abb: Thioredoxin Reductase 1(TXNRD1) (Created with BioRender.com).

Disulfidptosis, a recently discovered form of programmed cell death, has rapidly emerged as a research hotspot in tumor therapy due to its unique molecular mechanisms and selective sensitivity in specific tumor cells, offering potential novel intervention pathways for cancer treatment (Table 3, Fig. 4). Current research primarily focuses on inducing disulfidptosis specifically in tumor cells through various strategies to enhance therapeutic efficacy and overcome drug resistance [59,109–111].

**Table 3:** Strategies for targeting disulfidptosis in tumor therapy.

Type of Strategy	Specific Targets/Inducers	Mechanism of Action	References
Metabolic Targeting	GLUT1 inhibitors (BAY-876, KL-11743)	Block glucose uptake, leading to NADPH depletion and triggering actin cytoskeleton cross-linking	[68]
	G6PD inhibitors (6-Aminonicotinamide, RRx-001)	Reduce NADPH production and induce disulfidptosis	[30]
	TXNRD1 inhibitors	Induces intracellular cystine accumulation and disulfide bond deposition	[81]
Drug Repurposing	Nanodrug delivery systems (e.g., nanoparticles loaded with GOx)	Selectively blocks tumor glucose metabolism, disrupts tumor metabolism, and reduces damage to normal tissues	[114]
Precision Delivery	Disulfidptosis-related lncRNA/DRGs scoring	Guiding personalized chemotherapy and predicting drug sensitivity	[111,115]

Note: Abb: Thioredoxin Reductase 1 (TXNRD1), Glucose Oxidase (GOx).

### 5.1 Metabolic Pathway Targeting Strategies

The occurrence of disulfidptosis is highly dependent on intracellular NADPH levels and abnormal disulfide bond accumulation. Therefore, disrupting key metabolic pathways is a core strategy for inducing this form of cell death.

GLUT1 inhibitors (e.g., BAY-876, KL-11743): By blocking glucose uptake, they deplete NADPH in SLC7A11-high cancer cells, leading to abnormal disulfide cross-linking of actin cytoskeletal proteins and ultimately triggering disulfidptosis. Both *in vitro* and *in vivo* mouse studies have confirmed that these inhibitors significantly suppress the growth of SLC7A11-high tumors while exhibiting low toxicity to normal tissues [68].

G6PD inhibitors (e.g., 6-Aminonicotinamide, RRx-001): By suppressing the activity of G6PD, a key enzyme in the pentose phosphate pathway, they reduce NADPH production and thereby induce disulfidptosis, demonstrating potential therapeutic value in various cancer models [30].

TXNRD1 inhibitors: Target thioredoxin reductase 1, disrupt cellular redox homeostasis, cause excessive cystine accumulation and disulfide deposition, selectively kill cancer cells, and offer a novel drug target for clinical applications [81].

### 5.2 Drug Repurposing and Delivery System Optimization

To enhance targeting and safety, researchers are exploring the modification of existing drugs or biologics using advanced delivery systems. Nanodrug delivery systems (e.g., nanoparticles loaded with glucose oxidase GOx) can selectively block glucose metabolism in tumor cells, mimicking glucose deprivation, inducing NADPH depletion and disulfidptosis. Nanomedicine-based delivery systems play a pivotal role in inducing disulfidptosis in tumor cells through precise design and multifunctional integration. Their central function lies in actively targeting the essential prerequisites of disulfidptosis. For instance, selective inhibition of SLC7A11 can efficiently block cystine uptake, thereby depleting glutathione synthesis at its source and inducing a cellular redox crisis. Alternatively, the delivery of glucose oxidase can directly catalyze glucose consumption within tumors, creating a “starvation” microenvironment. Moreover, intelligent nanocarriers can encapsulate specific prodrugs that, in response to the tumor microenvironment—such as high intracellular glutathione levels—consume large amounts of NADPH and cysteine, ultimately triggering lethal protein disulfide crosslinking [112]. These strategies can be applied individually or combined with other modalities such as photodynamic or photothermal therapy, simultaneously disrupting the cytoskeleton and activating immune responses, thereby offering a powerful new approach to overcome tumor drug resistance [113]. This strategy not only effectively disrupts tumor metabolism but also reduces damage to normal tissues, improving the therapeutic window [114].

### 5.3 Precision Medicine and Personalized Therapy

Combining biomarkers to guide treatment decisions can help achieve precise induction of disulfidptosis. Scoring models based on disulfidptosis-related lncRNAs or disulfidptosis-related genes can be used to predict patient sensitivity to specific targeted drugs. For instance, ACTN4 expression levels may influence the efficacy of BRAF inhibitors, while MYL6 is associated with responses to MEK inhibitors, suggesting that such molecules could serve as potential biomarkers for guiding personalized chemotherapy [111,115].

## 6 Prospects

Disulfidptosis, as a newly defined mode of programmed cell death, not only expands our understanding of the cell death spectrum but also offers a highly promising metabolic intervention target for tumor therapy. Although current research has preliminarily uncovered its core molecular mechanism—driven by SLC7A11-dependent cystine uptake and NADPH depletion, leading to disulfide stress and actin cytoskeleton collapse—the field remains in its early stages, with many key scientific questions awaiting further exploration.

First, a specific biomarker system for disulfidptosis has yet to be established. Current screening criteria primarily rely on high SLC7A11 expression and glucose deprivation sensitivity, but the lack of dynamic, detectable molecular markers (such as specific protein disulfide modification profiles or redox state imaging probes) limits its precise identification and efficacy evaluation in clinical samples. Future efforts should integrate proteomics, redox proteomics, and single-cell sequencing technologies to systematically identify the unique molecular fingerprints of disulfidptosis and construct predictive models for patient stratification.

Second, the complexity of the regulatory network governing disulfidptosis is far from fully understood. Existing studies suggest the involvement of various factors such as LDHB, the WRC complex, the thioredoxin system, and endoplasmic reticulum stress, but the crosstalk mechanisms, spatiotemporal dynamics, and heterogeneity across different tumor types remain to be elucidated. Particularly critical is understanding how nutrient fluctuations, oxidative stress gradients, and immune cell interactions in the tumor microenvironment influence the threshold of disulfidptosis.

Moreover, therapeutic strategies targeting disulfidptosis face dual challenges of selectivity and resistance. Although GLUT inhibitors, G6PD inhibitors, and TXNRD1 antagonists have demonstrated antitumor activity in preclinical models, achieving tumor-specific induction while avoiding toxicity to normal tissues (especially high-metabolism organs) remains a central challenge in translational medicine. Nanodelivery systems, antibody-drug conjugates (ADCs), or conditionally activated prodrugs may offer technical solutions to this issue. Additionally, tumor cells may evade disulfidptosis by upregulating antioxidant pathways (e.g., NRF2, Trx system) or remodeling metabolic networks (e.g., enhancing glutamine metabolism), necessitating systematic research into resistance mechanisms to guide combination therapy strategies.

Finally, the synergistic effects between disulfidptosis and other forms of programmed cell death warrant in-depth investigation. Existing evidence suggests that systemic glucose deprivation can simultaneously induce disulfidptosis and ferroptosis, while nanodrugs can co-activate pyroptosis and disulfidptosis. Future research should systematically explore the synergistic mechanisms among multiple forms of cell death. For example, rational combination therapies could be designed to co-administer disulfidptosis inducers (such as GLUT1 inhibitors) with ferroptosis activators (such as GPX4 inhibitors), simultaneously targeting cancer cells from two metabolic dimensions to effectively prevent escape. In terms of clinical translation, the following priorities should be addressed: (1) Biomarker validation: prospectively validate SLC7A11 protein or mRNA levels as predictive biomarkers for patient stratification in early-phase clinical trials; (2) Drug delivery optimization: develop and refine stimulus-responsive nanoplatforms to achieve tumor-specific induction of disulfidptosis while minimizing systemic toxicity; (3) Combination therapy trial design: based on robust preclinical evidence, design clinical trial protocols combining disulfidptosis inducers with standard chemotherapy, targeted therapy, or immunotherapy.

In summary, disulfidptosis not only represents a novel paradigm of cell death but also reveals the fragile balance between metabolic dependency and structural stability in cancer cells. As mechanistic research deepens and technological advancements continue, targeting disulfidptosis is expected to become another key strategy in precision oncology, following apoptosis and ferroptosis, offering new breakthroughs in overcoming drug-resistant cancers.

## Data Availability

Data sharing not applicable to this article as no datasets were generated or analyzed during the current study.

## Abbreviations

ARE: Antioxidant Response Element
ATF4: Activating Transcription Factor 4
CASC8: Cancer Susceptibility Candidate 8
CCNB2: Cyclin B2
CRC: Colorectal Cancer
Dis score: Disulfidptosis Score
DPP7: Dipeptidyl Peptidase 7
F-actin: Filamentous Actin
FTO: Fat Mass and Obesity-associated protein
G6PD: Glucose-6-Phosphate Dehydrogenase
GBM: Glioblastoma Multiforme
GBMLGG: Glioblastoma and Lower Grade Glioma
GC: Gastric Cancer
GLUT1: Glucose Transporter 1
GPX4: Glutathione Peroxidase 4
GSH: Glutathione
GYS1: Glycogen Synthase 1
HBV-HCC: Hepatitis B Virus-related Hepatocellular Carcinoma
HCC: Hepatocellular Carcinoma
HMOX1: Heme Oxygenase 1
HNSC: Head and Neck Squamous Cell Carcinoma
KIPAN: Kidney Pan-cancer (Kidney Renal Clear Cell Carcinoma, Kidney Papillary Cell Carcinoma, and Kidney Chromophobe)
LAML: Acute Myeloid Leukemia
LDHB: Lactate Dehydrogenase B
LGG: Lower Grade Glioma
LIHC: Liver Hepatocellular Carcinoma
lncRNA: Long Non-coding RNA
LUAD: Lung Adenocarcinoma
m6A: N6-methyladenosine
MA: Meclofenamic Acid
MESO: Mesothelioma
MSI: Microsatellite Instability
NADPH: Nicotinamide Adenine Dinucleotide Phosphate (reduced form)
NRF2: Nuclear Factor Erythroid 2-Related Factor 2
OCCC: Ovarian Clear Cell Carcinoma
PAAD: Pancreatic Adenocarcinoma
PCa: Prostate Cancer
PDAC: Pancreatic Ductal Adenocarcinoma
PPARγ: Peroxisome Proliferator-Activated Receptor Gamma
PPP: Pentose Phosphate Pathway
Rac1: Ras-related C3 botulinum toxin substrate 1
RCD: Regulated Cell Death
ROS: Reactive Oxygen Species
SLC7A11: Solute Carrier Family 7 Member 11
Tex: T cell Exhaustion
TMB: Tumor Mutational Burden
UVM: Uveal Melanoma
WRC: WAVE Regulatory Complex
